# Tool to visualize and evaluate operator proficiency in laser hair-removal treatments

**DOI:** 10.1186/1475-925X-13-40

**Published:** 2014-04-08

**Authors:** Seungwoo Noh, Woo Seok Koh, Hyoung-woo Lim, Chiyul Yoon, Youdan Kim, Jin Ho Chung, Hee Chan Kim, Sungwan Kim

**Affiliations:** 1 Interdisciplinary Program for Bioengineering, Seoul National University, Seoul 110-744, Korea; 2 JMO Dermatology, Seoul 135-887, Korea; 3 Department of Mechanical and Aerospace Engineering, Seoul National University College of Engineering, Seoul 151-742, Korea; 4 Department of Dermatology, Seoul National University Hospital, Seoul 110-744, Korea; 5 Department of Biomedical Engineering, Seoul National University College of Medicine, Seoul 110-799, Korea; 6 Institute of Medical and Biological Engineering, Seoul National University, Seoul 151-742, Korea

**Keywords:** Laser visualization, Performance evaluation, Photomedicine, Simulation bed, Training tool

## Abstract

**Background:**

The uniform delivery of laser energy is particularly important for safe and effective laser hair removal (LHR) treatment. Although it is necessary to quantitatively assess the spatial distribution of the delivered laser, laser spots are difficult to trace owing to a lack of visual cues. This study proposes a novel preclinic tool to evaluate operator proficiency in LHR treatment and applies this tool to train novice operators and compare two different treatment techniques (sliding versus spot-by-spot).

**Methods:**

A simulation bed is constructed to visualize the irradiated laser spots. Six novice operators are recruited to perform four sessions of simulation while changing the treatment techniques and the presence of feedback (sliding without feedback, sliding with feedback, spot-by-spot without feedback, and spot-by-spot with feedback). Laser distribution maps (LDMs) are reconstructed through a series of images processed from the recorded video for each simulation session. Then, an experienced dermatologist classifies the collected LDMs into three different performance groups, which are quantitatively analyzed in terms of four performance indices.

**Results:**

The performance groups are characterized by using a combination of four proposed indices. The best-performing group exhibited the lowest amount of randomness in laser delivery and accurate estimation of mean spot distances. The training was only effective in the sliding treatment technique. After the training, omission errors decreased by 6.32% and better estimation of the mean spot distance of the actual size of the laser-emitting window was achieved. Gels required operators to be trained when the spot-by-spot technique was used, and imposed difficulties in maintaining regular laser delivery when the sliding technique was used.

**Conclusions:**

Because the proposed system is simple and highly affordable, it is expected to benefit many operators in clinics to train and maintain skilled performance in LHR treatment, which will eventually lead to accomplishing a uniform laser delivery for safe and effective LHR treatment.

## Background

Approximately two decades after the emergence of the first FDA-approved laser, laser hair removal (LHR) treatment has become one of the most successful applications of lasers in medicine [1]. According to recent statistics from the American Society for Aesthetic Plastic Surgery (ASAPS), more than 1.2 million LHR procedures were performed in the U.S. during 2012, which were the third most frequent treatments in all cosmetic procedures and the second most frequent treatments for men [2]. The prevalence of LHR is assumed to be largely attributed to increased societal concerns about aesthetics, as well as its proven safety, efficacy, and greater simplicity than conventional epilation methods [3-5].

The idea of LHR is based on the theory of selective photothermolysis. This elaborates the differences in absorption rates of light energy between the hair follicle and tissue owing to the differences in chromophores [6]. Therefore, hair follicles can be selectively destroyed by exposing the laser to the target area without aiming at each follicle. According to more recent findings, the essence of photoepilation is not just the heat generation in the hair follicle but the conduction of heat to the hair stem cells, because the hair stem cell itself lacks an appreciable amount of chromophores and is located outside the outer root sheath of the hair follicle [7]. Therefore, the delivery of an appropriate dose of laser light is paramount for safe and effective photoepilation.

Failure in choosing the correct laser intensity level often causes side effects such as pigment alteration, blistering, and erythema owing to excessive heat generated in the tissue [8,9]. Insufficient delivery of laser light can also be problematic, leading to ineffective outcomes or even paradoxical hypertrichosis [10,11]. To minimize such side effects, many studies have been performed to determine the optimal set of laser parameters to treat various body sites and skin types of patients [12-15].

Even with the right intensity of laser light, however, the actual amount of energy delivered to the target presents local variations when the laser treatment spots are not evenly applied [16-18]. The potential threat of side effects from this nonuniformly delivered laser light is serious when we consider that these treatments are often delegated to nonphysicians or even nonmedical personnel who lack sufficient training [2,19]. Physicians must also practice or train on new laser devices, new applicator tips with different dimensions, and different settings of laser parameters [20].

Therefore, it is highly desirable to develop an affordable system to evaluate the operator’s procedural performance in LHR treatments. In these cases, the spatial patterns of delivered laser light energy must be quantitatively accessed, but little attention has been focused on this issue thus far.

The difficulty in implementing such a system is the visualization of the irradiated area. Because infrared is used and no prompt marks are left on the treated area after LHR treatments, the irradiated spots cannot be easily traced. Recently, a group of researchers proposed a thermovision camera-based laser-visualizing method [21]. They successfully viewed the thermal changes in the skin and quantitatively analyzed the degree of overlaps and omissions. However, the use of an infrared camera is too expensive for general use. Moreover, it is only applicable to the postoperative assessment of performance accuracy and can be potentially erroneous when any type of skin-cooling mechanism is used. Other researchers utilized photosensitive paper and a camera to visualize the laser light [22] for investigating intensity profiles across a single spot to analyze the nonideal characteristics of an LHR device. However, this methodology cannot be directly applied to the LHR performance evaluation system, primarily because photosensitive papers are not reusable and extra steps are required to digitize the results. Some commercial devices incorporate an auxiliary visible laser light to guide an operator for aiming the position of the laser spot; however, these are inapplicable to contact-type LHR devices and the trajectory of the irradiated laser spots cannot be traced.

In this research, a relatively simple system is proposed to visualize and analyze the delivery patterns of laser sources during a simulated LHR procedure. The system is intended for preclinical uses to evaluate the proficiency of operators and features affordability and simplicity, based on an off-the-shelf PC camera and digital image processing methods. The purpose of this study is: 1) to demonstrate that the proposed system can quantitatively reflect the performance level of LHR treatments, and 2) to test the applicability of the system in the field by training novice operators and comparing two different treatment techniques (sliding versus spot-by-spot). For these purposes, six novice operators are recruited and their performance is evaluated according to four performance indices during the simulated procedures of LHR.

## Methods

### Experimental apparatus

In the experiments, commercially available laser equipment, a PC camera, and polarizers were used with a simulation bed built in-house. Table 1 summarizes the specifications of the devices.

**Table 1:** Summarized specifications of the devices used in the experiments

**Devices**	**Manufacturer**	**Size**	**Others**
Laser equipment	LightSheer XC, LUMENIS, Inc.	20 × 20 mm^2^ (applicator tip) 12 × 12 mm^2^ (laser window)	Fluence: 10–100 J/cm^2^ Repetition: 1–2 Hz Wavelength: 800 nm
Simulation bed		400 × 250 × 250 mm^3^	Made of 15 mm × 15 mm aluminum profiles
Silicon layer	Anonymous	400 × 250 mm^2^	To mimic the skin friction
Glass layer	Anonymous	400 × 250 mm^2^	To mechanically support the silicon layer
Mirror	Anonymous	400 × 350 mm^2^	To reflect the laser from the top to the front
Camera	SPC-A30M, Samsung, Inc.	50 × 50 × 70 mm^3^ (approx.)	Sensitivity: Visible and infrared regions Frame rate: 30 Hz Resolution: 640 × 480 pixels
Polarizers	Visible linear polarizing film, Edmund Optics, Inc.	Six orthogonally aligned 15 × 15 mm^2^ film cuts	transmission: > 40% at 800 nm

**Figure 1:**
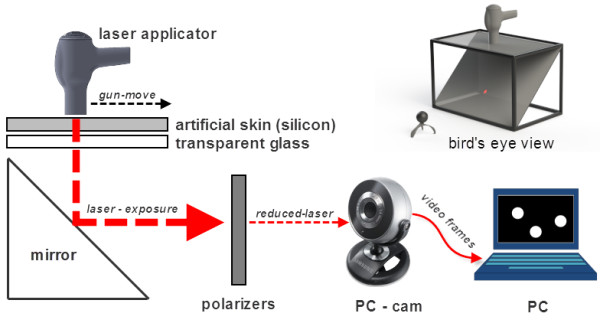
**Simulation bed used in this experiment.** Lasers emitted on the human skin layer were reflected to an infrared camera. A silicon rubber sheet was stacked on top of the simulation bed to mimic the friction of human skin and a normal PC camera was utilized for the visualization of the laser.

The simulation bed was made of aluminum profiles in a cuboid shape that was open on each side. To mimic the friction of human skin during LHR simulation, a sheet of semitransparent silicon rubber was placed on transparent glass on the top of the simulation bed. Inside the simulation bed, an angled mirror was installed (at 45 degrees) to reflect the laser light coming from the top to the front of the simulation bed. Figure 1 shows the simulation bed with the positioned camera.

A PC camera (SPC-A30M, Samsung, Seoul, Korea) was placed at a distance of 200 mm from the front side of the simulation bed to capture the laser light. Its position and viewing direction were adjusted to fully view the treatment area reflected in the mirror and to not distort the scene. By attaching a series of polarizers (visible linear polarizing film, Edmund optics, Barrington, NJ) in front of the camera lens and taking advantage of the PC camera’s sensitivity to the infrared lights, we could make it function as an infrared camera. Additionally, the polarizers protect the camera by significantly attenuating the intensity of input laser light and increasing the signal-to-noise ratio of the video images by removing background noises. Because the intensity of the laser light is much higher than visible-band light, the captured video images only contain the laser spot. Ideally, two orthogonally aligned polarizers can block all of the incoming lights. However, owing to practical discrepancies, six layers of polarizers were used in this experiment.

**Figure 2:**
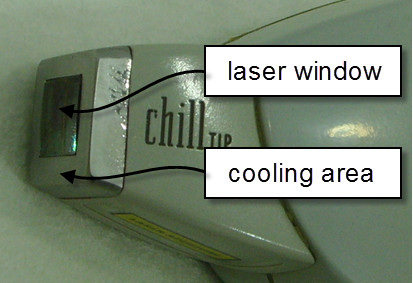
**Applicator tip of the laser device used in the experiments.** The size of the applicator tip is larger than the laser window owing to the cooling area located at its perimeter.

Contact-type diode laser equipment (LightSheer XC, LUMENIS, Santa Clara, CA) was used in the experiments. The device fires a single pulse of laser light when the trigger button on the laser applicator is pressed or a train of continuous pulses at a predetermined rate when the button is held. The fluence and repetition frequency of the laser are configurable, but were set to 25 J/cm2 and 2 Hz, the most common settings used in clinics, during all experiments. The size of the applicator tip was larger than the size of the laser window owing to the cooling area located at its perimeter (Figure 2).

### Study design

Six novice operators who had no prior knowledge about the LHR procedure were recruited (all men, 24–31 years) and simulated LHR treatment on the top of the simulation bed. Operators were asked to achieve a delivery as uniform as possible of the laser on a rectangular target area designated as 140 mm × 90 mm. The usage of gel was mandatory to simulate the real conditions of an LHR procedure. Prior to the experiments, a didactic lecture was given by an experienced dermatologist about the safety issues, principles, and techniques of LHR treatment. A real demonstration followed the lecture.

Two different techniques of treatments, spot-by-spot (SBS) and sliding, which are most frequently used in clinics, were simulated. In the SBS technique, a single laser pulse was fired at a time while the laser applicator was repeatedly placed on and removed from the skin by an operator. In the sliding technique, laser pulses were continuously fired while the laser applicator was slid, maintaining contact with skin throughout the whole procedure. The order of the simulated techniques was randomly assigned to avoid the possible effect of habituation to the experimental apparatus.

**Figure 3:**
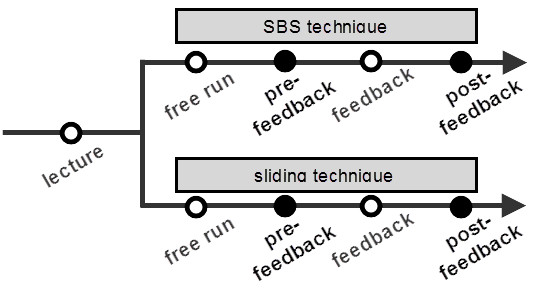
**Design of simulation experiments.** Two different treatments techniques were simulated and each treatment technique was composed of two recorded sessions of the simulation (filled circles). Feedback on the procedural performance was given between these two successive sessions.

Each simulated technique of treatment was composed of two separate sessions; the infrared camera installed inside the simulation bed recorded all simulated sessions. To simulate the training of novices, feedback on the procedural performance was given to the operator between the two sessions. Feedback was provided by viewing the image-processed video recorded during the prefeedback session, and erroneous laser delivery patterns such as overlapping and omissions of laser spots were confirmed automatically. Before initiating each session, operators were allowed free time to test and become accustomed to their strategy of treatment. Time for the free run was unlimited and varied among operators, ranging from 1 to 3 min. Figure 3 shows the overall study design.

This experimental protocol was approved by a local institutional review board (IRB No. C-1302-075-467 at Seoul National University Hospital) and conducted in accordance with the principles of the 2004 version of the Declaration of Helsinki. All subjects signed the informed consent form.

### Image processing

**Figure 4:**
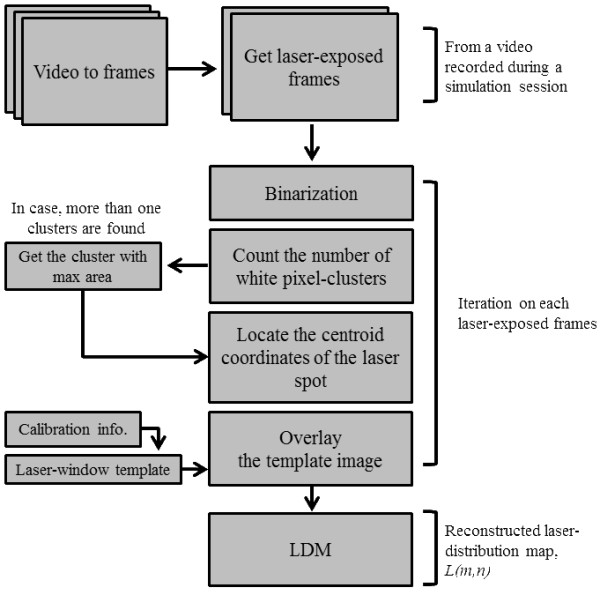
**Block diagram of image processing steps to synthesize an LDM.** Laser-exposed frames were extracted from the video recorded during a simulation session. Each laser-exposed frame was binarized to clearly detect the laser spot, then its centroid position was computed. The LDM was reconstructed by overlaying the template image of the laser window to each centroid position of the detected laser spot.

A laser energy distribution map (LDM) was synthesized through a series of image processing algorithms on recorded video frames. Figure 4 shows the block diagram of the overall image processing steps.

First, calibration between physical and image space was made by locating four corners of the target area with 10 laser exposures. The calibration process revealed that 140 mm of physical dimension was equivalent to 397 pixels in the image space. The degree of distortion in the camera view and the errors in locating the laser spots were inconsequential.

**Figure 5:**
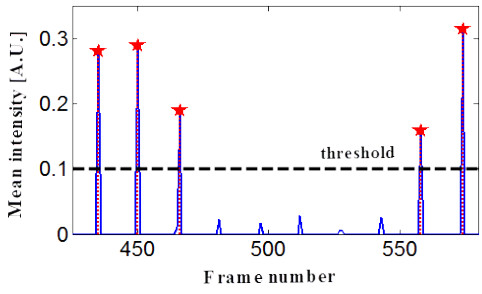
**Mean intensity plot of the recorded video to specify laser-exposed frames.** Laser-exposed frames were extracted from the video based on the brightness scale of frames. Peaks in the plot represent laser-exposed frames; however, some peaks with less than 30% of normal peak height were not counted as valid laser-exposed frames. These frames occurred when the laser was fired in the air, mostly during directional changes of the laser applicator when the SBS technique was simulated.

Next, the frames containing single pulses of the laser were retrieved from the video recording of a simulated session. Because a laser spot is shown as a cluster of bright pixels in the image, the laser-exposed frames can be selected from a plot of mean grayscale of each image frame. As exemplified in Figure 5, peaks in the plot represent laser-exposed frames. Some frames were not counted as valid laser-exposed frames; for example, those with no laser spot but an overall increase in brightness level at less than 30% of normal laser-spotted frames. These frames occurred when the laser was fired in the air, mostly during directional changes of the laser applicator during the simulated SBS technique.

**Figure 6:**
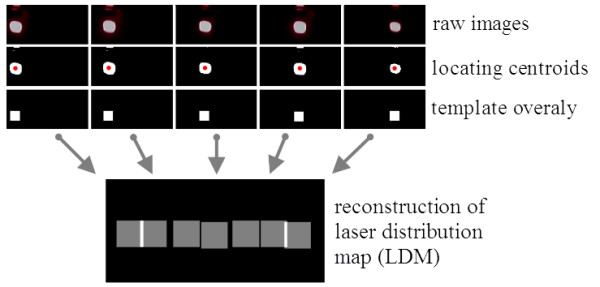
**Illustration of LDM synthesis by locating laser spots and overlaying template images.** The raw images of laser spots were binarized and a square template mimicking the actual size and shape of the laser-emitting window was overlaid to the centroid position of each laser spot by allowing superimposition. The reconstruction process was necessary because distortions were evident in the raw images, caused by the scattering effect of the rubber sheet and the point spread characteristics of the camera system.

Next, the position of the laser spot in each laser-exposed frame was located (Figure 6). Images were converted to black and white (binarization) with a threshold of 30% of maximum value in the frame. Then, the coordinates of the centroid of a white cluster were computed. Normally, the converted images contained only a single cluster of white pixels. However, in some cases, the reflected laser light from the aluminum profile appeared as additional white clusters. In such cases, the cluster with the largest area was regarded as the true laser spot.

Finally, a square template that represents the actual shape and size of a laser spot was overlaid at the position of the detected laser spot (Figure 6). The gray value of the pixels inside the template was set to a constant of 1, which reflects a uniform distribution of the energy delivered from the laser light. This type of reconstruction process was necessary because the captured images of laser spots were blurred owing to the scattering effect of the silicon rubber sheet as well as the point spread characteristics of the camera system. A calibration process determined the size of the template to be 34 × 34 pixels. The LDM was built by accumulating all of these template-represented laser spots in the retrieved frames, allowing superimposition at overlapped regions (multiple doses of laser). Each pixel in the target area was initially set to zero and accumulated its value by the gray value of the template every time it was attributed to a laser spot.

### Performance indices

For the quantitative analysis of the LDM, four performance indices are proposed by using general statistics. The first two indices, δ_0_ and δ_z_, are measures of the errors in the LDM, defined as the percent ratio of untreated and redundantly treated areas to the total area of the target, respectively, which is shown in Eqns. 1 and 2.

(1) $$\delta_{0}\left[\%\right]=\frac{\left(A_{0}-A_{1}\right)}{A_{0}}\times 100$$

(2) $$\delta_{z}\;\left[\%\right]=\frac{\sum_{k=2}^{\alpha }A_{k}}{A_{0}}\times 100$$

Where

(3) $$A_{k}=\overset{M}{\underset{m=1}{\sum }}\overset{N}{\underset{n=1}{\sum }}L\left(m,n\right)○k\;k=1,2,\ldots ,\alpha \;\mathrm{and}\;A_{0}=M\times N$$

(4) $$x○y=\left\{\begin{array}{c} 1,\mathrm{if}\;x\geq y\\ \;0,\text{otherwise} \end{array}\right)$$

Here, α is the highest pixel value found in the LDM, L(m,n), which denotes the maximum redundancy in the laser delivery. M and N represent the dimensions of the image in pixels (number of rows and columns, respectively). The maximum value of δ_z_ may exceed 100% because the target area can be treated redundantly with more than two laser exposures (multiple doses are counted by summing each A_k_).

The μ is an index that represents an operator’s estimation of the spot size, which is defined by using the mean of every two consecutive spot distances, d_c_, as depicted in Eqn. 5.

(5) $$\mu \;\left[\mathrm{mm}\right]=\text{mean}\left(d_{c}\right)\times C$$

where

(6) $$d_{c}=\;\left\{x_{i}|\;x_{i}=|\;S\left(i\right)-S\left(i+1\right)\;|,i=1,2,\ldots ,\left(\beta -1\right)\;\right\}$$

The constant C is the conversion ratio between the physical and image spaces, which was found to be 0.35 mm/pixel in in the calibration step of our experiments. S is the array containing the centroid of the position of each laser spot. β is the number of laser spots exposed to the target. The ideal value of μ is equal to the actual size of laser window (which is 12 mm in this study).

The υ is the measure of randomness in the LDM. It is formulated as a normalized form of distance variations measured from each laser spot to its nearest one:

(7) $$\upsilon \;\left[\%\right]=\frac{\text{std}\left(d_{n}\right)}{\text{mean}\left(d_{n}\right)}\times 100$$

where

(8) $$\begin{array}{l} d_{n}=\left\{y_{i}\left|y_{i}=\text{min}\left(\left|S\left(i\right)-S\left(j\right)\right|\right)\right)\right\},\;\forall j\in \left\{1,2,\ldots ,\beta \right\} \end{array}$$

An increase in υ may result in increases of both or either δ_0_ and δ_z_; however, the reverse is not always true.

**Figure 7:**
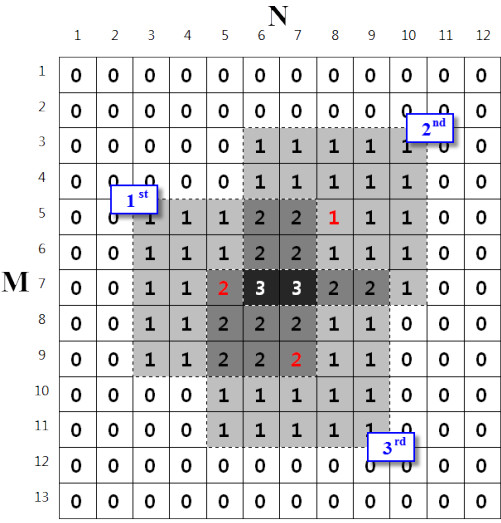
**Synthetic LDM to illustrate the computation process of performance indices.** The LDM has three overlapped laser spots and the pixel values indicate the amount of laser exposure at the site. Here, the maximum redundancy in laser delivery is 3 and the centroid position of each laser spot was marked as red. The indices δ_0_ and δ_z_ are computed based on the pixel values, and μ and υ are computed based on the centroid position of laser spots.

To illustrate the computation of performance indices, a synthetic LDM with three shots of lasers is shown in Figure 7. In this example, the size of the LDM and the laser spot were set to 13 × 12 and 5 × 5 pixels and the conversion ratio, C, was set to 1 (i.e. 1 mm = 1 pixel). The numbers in the pixels indicate the amount of laser exposure at the site, and the centroid position of each laser spot is marked in red. The computation process is as follows:

1. Maximum redundancy in the laser delivery α = 3.

2. A_0_, A_1_, A_2_, and A_3_ are 156, 58, 15, and 2, according to Eqn. 3.

3. δ_0_ = 62.82% and δ_z_ = 10.90%, according to Eqns. 1 and 2.

4. S = {(7, 5), (5, 8), (9, 7)}.

5. d_c_ = {3.61, 4.12} and d_n_ = {2.83, 3.61, 2.83}, according to Eqns. 6 and 8.

6. μ = 3.87 mm, υ = 0.15%, according to Eqns. 5 and 7.

### Statistical comparisons

A total of 24 LDMs (six subjects, two techniques for treatments, and pre/postfeedback) were collected for the statistical comparison.

To validate the efficacy of proposed indices in assessing the performance of the LHR, an experienced dermatologist sorted the collected LDMs into three groups according to performance. Eight LDMs that presented the highest level of performance were selected first and designated as group G. The other 16 LDMs presented relatively poor performance compared to group G, but were sorted again into groups P_+_ and P_-_ because there were alternative reasons for poor performance. Therefore, LDMs having too many overlaps were assigned to group P_+_ and those with too many omissions to group P_-_, with eight LDMs each.

The effect of the training was examined by comparing data between prefeedback and postfeedback sessions. The comparison was made for each treatment technique by comparing D1 and D2 for SBS technique and D3 and D4 for the sliding technique. Because we also hypothesized that there is a difference between treatment techniques, D1 was separately compared to D3 and D2 to D4, with the presence of feedback.

Statistical analysis of the experimental data was performed by using the Student’s t-test for the matched paired, one-sample t-test, one-way ANOVA, and a Turkey post-test at a significance level of 0.05. Prior to the t-test and ANOVA, the normality of the data was assessed by using Shapiro-Wilk’s method.

## Results

### Validation of performance indices

**Figure 8:**
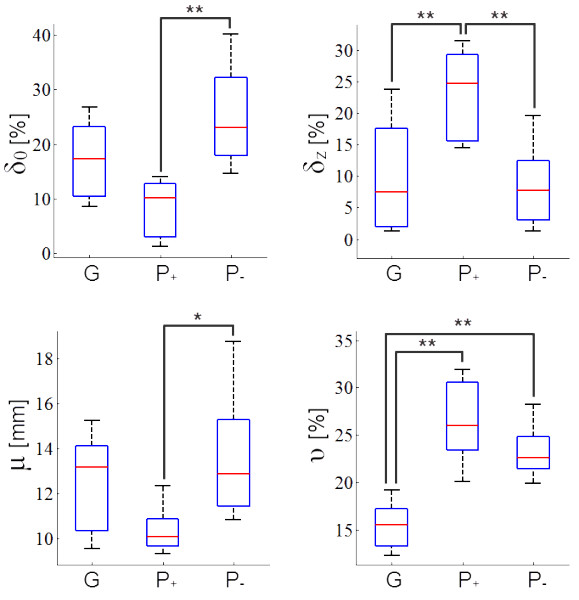
**Differences in indices among performance groups.** Each performance group was characterized by using a combination of indices (*P < 0.05, **P < 0.01). The quantitative description of satisfactory patterns in laser delivery in LHR treatment aims to achieve minimal randomness in the spatial distribution of laser spots, with accurate estimation of the size of the laser spots.

The results of ANOVA indicated that the mean values of performance indices are significantly different among performance groups (Figure 8). Group G showed lower values of δ_z_ and υ than group P_+_. The υ value of group G was also lower than that of group P_-_. Group P_+_ and P_-_ could be distinguished by using any single performance index except for υ. The one-sample t-test result for μ showed that only group P_+_ presented a significantly different value of μ from the actual laser window size of 12 mm (Table 2).

**Table 2:** Deviation of μ from actual laser window size

	**μ: 12 mm**	
	**(mean ± SD)**	***P***
Group G	+0.50 ± 2.17	0.537
Group P_+_	-1.65 ± 2.1	0.002**
Group P_-_	1.59 ± 2.74	0.144

**P < 0.01 Negative value indicates shorter spacing between consecutive laser spots.

**Figure 9:**
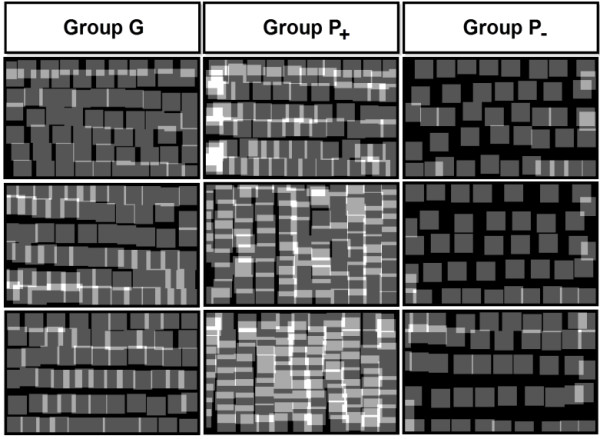
**Comparison of LDMs that represent different levels of performance.** Nine LDMs selected from three performance groups are shown to exemplify different levels of performance. The LDMs from group G presented better laser distribution than the rest. Specifically, group P_+_ and group P_-_ exhibited more overlapping and omission, respectively, than group G.

To exemplify different levels of performance, nine LDMs selected from three performance groups are shown in Figure 9. It is clear that the LDMs from group G presented better laser distribution than the rest. Specifically, groups P_+_ and P_-_ exhibited more overlapping and omission, respectively, than group G. In these cases, the mean value of δ_z_ was 29.08 for group P_+_ and 6.05 for group G. The value of δ_o_ was 23.77 for group P_-_ and 13.12 for group G. Therefore, the quantitative analysis results are consistent with the results of visual inspection.

### Training of operators

Four categories of data described in Table 3 were considered to study the effects of training (prefeedback versus postfeedback). The mean values in Table 4 represent the differences between D1 and D2 for the sliding technique and between D3 and D4 for the SBS technique.

**Table 3:** Categories of collected data for statistical comparison

**SBS technique**		**Sliding technique**	
**Prefeedback**	**Postfeedback**	**Prefeedback**	**Postfeedback**
D1	D2	D3	D4

**Table 4:** Effects of feedback depending on treatment technique

	**Sliding mode**		**SBS mode**	
	**(mean ± SD)**	***P***	**(mean ± SD)**	***P***
δ_z_ [%]	+6.00 ± 8.03	0.127	+1.07 ± 5.27	0.641
δ_0_ [%]	-6.32 ± 5.12	0.029*	-4.68 ± 6.14	0.121
μ [mm]	-1.79 ± 1.67	0.047*	-0.32 ± 1.08	0.504
υ [%]	-0.77 ± 3.83	0.645	+0.75 ± 1.22	0.188

Prefeedback μ values were >12 mm, regardless of techniques.

(13.49 in sliding, and 13.86 in SBS).

Positive signs indicate a higher index value in postfeedback session.

*P < 0.05.

The paired t-test results of the training indicate that the feedback was effective when the sliding technique was used, as indicated by the reduction in δ_0_ and μ. The value of μ decreased from 13.49 mm to 11.70 mm, approaching the ideal value of 12 mm. The increase in δ_z_ was not desirable, but the degree of its change was not statistically significant. In contrast to the sliding technique, none of the performance indices changed meaningfully when the SBS technique was used. The values in Table 4 represent the differences between the indices in the postfeedback session computed relative to the prefeedback session.

### Comparison of treatment techniques

To compare the two different treatment techniques, the differences between D1 and D3 were computed for the case of prefeedback. The same procedure was followed between D2 and D4 for the case of postfeedback. The results are summarized in Table 5. During prefeedback sessions, operators showed a higher value of υ when the sliding technique was used than when the SBS technique was used. The remaining indices did not show differences between the treatment techniques. The differences were measured relative to the SBS technique.

**Table 5:** Results of LDM analysis on the effects of treatment technique

	**Prefeedback**		**Postfeedback**	
	**(mean ± SD)**	***P***	**(mean ± SD)**	***P***
δ_z_ [%]	-2.87 ± 8.50	0.445	+2.06 ± 12.80	0.710
δ_0_ [%]	+5.40 ± 6.78	0.108	+3.76 ± 13.31	0.520
μ [mm]	+1.63 ± 1.58	0.053	+0.16 ± 1.45	0.794
υ [%]	+7.21 ± 5.79	0.028*	+5.69 ± 5.79	0.146

Positive signs indicate higher index values in the sliding technique.

*P < 0.05.

## Discussion

As pointed out in a previous study [23], methods to learn clinical skills are changing as the opportunities for learning through work with actual patients has diminished. The same holds true for LHR treatment because current residency programs in dermatology place insufficient emphasis on photodermatology and laser therapy [24], and even nonphysician treatments have become prevalent [25]. The absence of validation methods is a problem in LHR treatment; because of this, no operators, even licensed ones, can initiate treatment with great confidence. The use of models or simulators has been the common practice to tackle this shortage of experience [23].

The proposed LHR evaluation tool successfully visualized simulated patterns of laser delivery and evaluated them in terms of four indices. Significant differences in indices among performance groups were found; however, none of these indices were sufficient to characterize all of the performance groups at once. Therefore, the proposed indices should be used in combination to assess the level of performance.

The ideal situation of group G, the most well-performing group, should have a μ value of 12 mm and the lowest values in the rest of the indices. However, only the two indices, μ and υ, corresponded to this expectation. Therefore, the quantitative description of satisfactory patterns in laser delivered LHR treatment aims to achieve minimum randomness in the spatial distribution of laser spots and accurate estimation of the size of laser spots. A moderate level of omissions and overlapping appears to be allowed; however, it has been deduced that the degree of overlapping is a more important factor than that of omissions when describing satisfactory performance, because group G could be distinguished from one of the poorly performing groups in terms of δ_z_ but not δ_0_.

**Figure 10:**
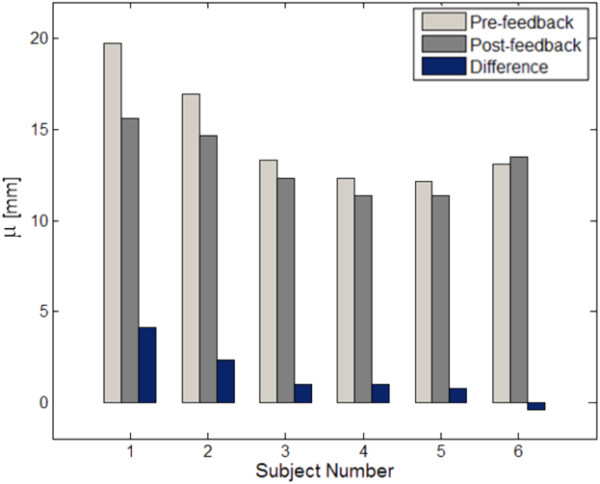
**Individual changes of μ after training for the sliding technique.** The greater the deviation an operator exhibited in μ from the ideal value of 12 mm, the greater improvement was observed after training, except for subject 6.

The training was effective only when the sliding technique was used, as indicated by the improvements in δ_0_ and μ values (Table 4). A careful examination of the changes in μ values is given in Figure 10. The more deviation an operator exhibited in μ from its ideal value of 12 mm, the more improvements were observed after training. The reduced δ_0_ after the training is believed to be the result of a more accurate μ. In contrast to the sliding technique, operators’ performance did not significantly improve when using the SBS technique. The difference of training effects between treatment techniques is hypothesized to be largely attributable to the characteristics of the gel. In the SBS technique, gels bear the marks of previous contact with the applicator tip at every departure of the applicator tip from the target surface. Operators may have been misled by the illusion of marks that inhibited them to reflect on the feedback from the system. This was confirmed by interviewing the operators as a group after finishing the experiments. Five of six operators replied that they took advantage of the marks on the gel to easily determine the position of the laser spot during the simulation of the SBS technique.

In the sliding mode, however, operators were not deceived by marks on the gel, because an operator had to adaptively control the amount of force applied to the laser applicator tip to cope with the varying frictions while maintaining contact with the surface. The results listed in Table 5, which compare treatment techniques, support this hypothesis. The higher value of υ in the sliding technique indicates that the operators experienced more difficulties maintaining constant distances between successive laser spots while using this technique, particularly before feedback was provided. This result also supports the reason for recommending the SBS technique to novice operators in clinics. Therefore, operators are required to be well-informed about the effects of the gels on performance, depending on the technique used for the LHR treatment.

It was interesting to note the trend of elevated prefeedback μ values, regardless of treatment techniques (Table 4). This indicates that the operators overestimated the size of the laser spot, which is assumed to be attributable to the mismatch in size between the applicator tip and the laser window. Because the actual size of the laser-emitting window was smaller than that of the applicator tip, and only the top view of the applicator tip was visible during the treatment, operators were susceptible to overestimating the size of the laser spot.

The presented study has three limitations, described as follows. First, the aforementioned criterion of satisfactory delivery of the laser in LHR treatment can be prematurely generalized, because the performance groups presented in this paper reflect only a single professional dermatologist’s opinion. More general wisdom—for example, a composite score of LHR proficiency—will be found when additional tests are conducted with a group of dermatologists by comparing their simulated results against those of novices. Second, owing to the practical limitations in utilizing clinical settings, the system was tested by a small number of operators; therefore, the statistical analysis performed in this study may have a high probability for type II error. In other words, the performance indices might be more significant for characterizing performance groups and observing the effects of training. Additionally, a prospective study might be conducted to confirm the long-term efficacy of the system in operator training; however, the usage of simulators has proven effectiveness in other medical fields for the acquisition of particularly junior levels of maneuverability [23]. Third, the system might not be applicable to noncontact types of LHR devices, so the simulation of treatments on curved areas of the body would not be feasible. Because a mirror was used to reflect the laser beam to the camera, the location of the laser spot can be erroneous as the laser applicator deviates from the vertical line to its target surface. However, this limitation does not seriously degrade the fidelity of the simulation. Referring to the results from a research group that recently analyzed patients’ thermal deposit images during cosmetic laser procedures [21], the patterns in experts’ laser delivery (Figure 7 in the study [21]) resembles those of well-performing groups in our experiments (Figure 9 in this experiment). In the previous study, operators also tried to cover the target area with uniform but not overlapping laser spots, as in our experiments. Although the percent errors of the omitted and overlapped areas were not directly comparable owing to some differences in the methods used for computing indices, the similarities between the simulated and clinical trials ensure the applicability of the proposed system in operator training. The pulse rate of the laser is another aspect of hardware limitation. A normal webcam can capture up to 30 frames of images per second; therefore, lasers pulsed higher than this rate may not be correctly detected. However, such settings are uncommon in clinical practice.

Even with these limitations, it is expected that the proposed system could be effectively utilized in clinics, because it is a cost-effective and intuitive solution to visualize and evaluate the proficiency of LHR treatment. The system might be improved to be used in other areas of photomedicine. For example, laser applications in the treatment of pigmented lesions and facial rejuvenation require different degrees of laser overlaps and patterns, depending on the need of the patient and the treatment settings [26,27]. Therefore, the performance indices might be adjusted to reflect the general guidelines or an expert’s strategy for various treatments. An improvement to the software is also expected. Accounting for thermal relaxation time, rather than simply counting the number of redundant exposures, will be more advantageous in predicting actual thermal damage to the tissue.

## Conclusions

A uniform laser delivery during LHR treatment is significant for safe and effective treatment. A highly affordable system, which is also easy to operate, has been developed in this research. The proposed system was able to visualize and evaluate laser patterns during preclinical trials without using an expensive infrared camera. For the study, four useful performance indices were proposed for assessing the proficiency of operators during LHR treatment. With these indices, the developed system could quantitatively analyze an operator’s proficiency in LHR treatment. A performance analysis of the proposed, affordable system has shown that operators reduced omission errors by 6.32% and accurately estimated the spot distances to match the actual size of the laser-emitting window. Further, the proposed system was used as a scientific tool for the comparison of two different treatment techniques (sliding versus spot-by-spot) by observing the performances. Therefore, the proposed training system is expected to benefit many operators in clinical practice and to maintain skilled performance in LHR treatment, which may result in eventually accomplishing a uniform laser delivery treatment. The proposed system may also be applicable to other areas of photomedicine.

## Competing interests

The authors declare that they have no competing interests.

## Authors’ contributions

SN proposed an idea to visualize and analyze dermatologic laser treatments and played a leading role in preparing the manuscript. WSK, as a medical professional, has established a niche in the quantification of laser treatment and played a consultant role in designing the study. HL participated in designing the experimental apparatus and was involved in drafting the manuscript. CY participated in the study design and provided useful tips for performing statistical analysis. YK actively participated in drafting the manuscript to increase the integrity of the data. JHC played a consultant role in designing the study as a medical professional. HCK and SK conceived of the study, participated in its design and coordination, and helped to draft the manuscript. All authors read and approved the final manuscript.
